# The Positron Emission Tomography Tracer 3’-Deoxy-3’-[^18^F]Fluorothymidine ([^18^F]FLT) Is Not Suitable to Detect Tissue Proliferation Induced by Systemic *Yersinia enterocolitica* Infection in Mice

**DOI:** 10.1371/journal.pone.0164163

**Published:** 2016-10-04

**Authors:** Stefan Wiehr, Anna-Maria Rolle, Philipp Warnke, Ursula Kohlhofer, Leticia Quintanilla-Martinez, Gerald Reischl, Ingo B. Autenrieth, Bernd J. Pichler, Stella E. Autenrieth

**Affiliations:** 1 Werner Siemens Imaging Center, Department of Preclinical Imaging and Radiopharmacy, Eberhard Karls University Tübingen, Tübingen, Germany; 2 Institute of Medical Microbiology and Hygiene, Eberhard Karls University Tübingen, Tübingen, Germany; 3 Institute of Pathology, Eberhard Karls University Tübingen, Tübingen, Germany; 4 Department of Internal Medicine II, University Hospital Tübingen, Tübingen, Germany; 5 Institute of Medical Microbiology, Virology and Hygiene, University Medicine Rostock, Rostock, Germany; Biomedical Research Foundation, UNITED STATES

## Abstract

Most frequently, gram-negative bacterial infections in humans are caused by Enterobacteriaceae and remain a major challenge in medical diagnostics. We non-invasively imaged moderate and severe systemic *Yersinia enterocolitica* infections in mice using the positron emission tomography (PET) tracer 3’-deoxy-3’-[^18^F]fluorothymidine ([^18^F]FLT), which is a marker of proliferation, and compared the *in vivo* results to the *ex vivo* biodistributions, bacterial loads, and histologies of the corresponding organs. *Y*. *enterocolitica* infection is detectable with histology using H&E staining and immunohistochemistry for Ki 67. [^18^F]FLT revealed only background uptake in the spleen, which is the main manifestation site of systemic *Y*. *enterocolitica*-infected mice. The uptake was independent of the infection dose. Antibody-based thymidine kinase 1 (Tk-1) staining confirmed the negative [^18^F]FLT-PET data. Histological alterations of spleen tissue, observed via Ki 67-antibody-based staining, can not be detected by [^18^F]FLT-PET in this model. Thus, the proliferation marker [^18^F]FLT is not a suitable tracer for the diagnosis of systemic *Y*. *enterocolitica* infection in the C57BL/6 animal model of yersiniosis.

## Introduction

Molecular imaging with positron emission tomography (PET) allows for the determination of the metabolic and functional parameters of living cells [1] and has emerged as a rapid, non-invasive and highly sensitive approach to identify sites of infection and inflammation more rapidly than conventional laboratory-based diagnostic techniques [2]. These traditional diagnostic modalities often result in long turn-around times and clinicians have to treat patients empirically with broad-spectrum antibiotics until specific diagnostic results are available [3–4]. Infectious pathogens are a serious health issue, and their accurate detection remains a major challenge in medicine. Effective treatment often relies on pathogen identification at an early stage of infection, and yet many infections remain undiagnosed prior to systemic manifestation [5].

The gram-negative bacterium *Yersinia enterocolitica* (*Ye*) belongs to the family of Enterobacteriaceae and causes gastrointestinal as well as systemic infections with focal abscesses in mice and men mainly located in the spleen and liver [6]. The infection is transmitted by the ingestion of contaminated food or drinking water leading to severe diarrhea, enterocolitis, and mesenteric lymphadenitis [7].

The ^18^F-radiolabeled thymidine derivative 3’-deoxy-3’-[^18^F]fluorothymidine ([^18^F]FLT) has been used as a tool for the PET imaging of cancer in preclinical as well as clinical research settings for many years. There is an ongoing debate regarding the degree to which [^18^F]FLT PET quantitatively reflects tumor proliferation. This issue has been controversially discussed for more than a decade [8]. Many preclinical and clinical oncology studies have demonstrated varying correlations between the [^18^F]FLT uptake of tumors and histological markers of proliferation such as Ki 67 [8–14].

Previously, we could show that the PET glucose marker Fluorine-18 fluorodeoxyglucose ([^18^F]FDG), the major clinical PET tracer used for the detection of malignancies, is able to detect inflammation in the spleen of high dose *Ye*-infected mice, but not in low dose *Ye*-infected animals [15]. However, [^18^F]FLT is able to detect inflammation in arthritic ankles of glucose-6-phosphate-isomerase (GPI) serum-injected mice [16] and proliferation of carcinoma cells in subcutaneous CT26 mouse colon tumors [17]. While [^18^F]FLT is able to detect proliferation in these inflammation models, proliferation with this tracer has not been demonstrated in models of sterile inflammation [8, 18]. [^18^F]FLT uptake is associated with the salvage pathway, which typically provides DNA precursors to proliferating cells. A second mechanism, the *de novo* synthesis pathway, is also able to produce thymidine monophosphate for DNA integration in proliferating cells, but does not lead to uptake of [^18^F]FLT [8, 19]. Despite this, the mechanisms limiting [^18^F]FLT PET in the *de novo* thymidine pathway have yet to be determined[8]. Tissues with high proliferative potential, including lymphoid tissue, predominantly utilize the salvage pathway instead of the *de novo* pathway for DNA synthesis [20]. Our animal model for *Ye* infection is characterized by fast proliferation of the bacteria and a highly activated immune system with immune cells infiltrating the infected spleens of the mice [21]. The eradication of *Ye* is dependent on proliferating CD4+ T cells, thus we expected that [^18^F]FLT, specific for cell proliferation, is suitable for detection of *Ye* infection. Moreover, *Ye* highly proliferate, at least during high dose infection, and so we expected high Tk-1 activity associated with elevated [^18^F]FLT uptake in those infected organs. Furthermore, successful detection of localized bacterial infections with [^18^F]FLT has been previously reported in an animal model, where *Staphylococcus aureus* was implanted into the tibia of rabbits [22], and in a mouse model of *Salmonella typhimurium* infection in the thigh muscle [23]. Recently, Heinzmann et al. evaluated the relationship between endogenous thymidine concentrations and the uptake of [^18^F]FLT in preclinical tumor models and concluded that tumor thymidine concentrations were not correlated with [^18^F]FLT uptake in the models tested [24]. This work implies that endogenous thymidine concentrations in tumors alone is not sufficient to predict [^18^F]FLT uptake [24], which is line with the findings of McKinley et al. [8]. This conflicts with other studies, where endogenous thymidine in rodent’s serum can strongly affect the *in vivo* uptake of [^18^F]FLT in the target organs, further demonstrating the conflicting results of [^18^F]FLT PET imaging in small animals [18, 25]. Based on these findings, the aim of this study was to evaluate the suitability of [^18^F]FLT for its use in the detection of a *Ye* induced inflammation. A well-established murine model of yersiniosis was used for our experimental setup, which resembles the course of *Ye* infection in humans [26].

## Materials and Methods

### Mice and Infection

All animal procedures were performed according to protocols that were approved by the Regierungspräsidium Tübingen (Permit Number: IZ1/10) as per guidelines from the European Health Law of the Federation of Laboratory Animal Science Associations (FELASA). The animals were kept under standardized environmental conditions (20 ± 1°C room temperature, 50 ± 10% relative humidity, 12 h light-dark cycle) and received food and water *ad libitum*. Female C57BL/6JOlaHsd mice were infected with 5 x 10^4^ (high-dose, n = 8) or 1 x 10^3^ (low-dose, n = 7) colony forming units (CFU) of *Ye* WA-314 (serotype 0:8) in 200 μl of PBS via injection into the tail vein. PBS treated mice served as control group (n = 3). The health and general wellbeing of the mice were assessed daily, and body weights were measured daily. No adverse events occurred during the time course of 3 days of infection and no or marginal weight loss was observed in mice due to infection and/or anesthesia. All animals were sacrificed by cervical dislocation under deep anesthesia.

### [^18^F]FLT PET Tracer Production, PET/MR Imaging and *ex vivo* Biodistribution

Fluorine-18 was produced as [^18^F]fluoride at a PETtrace cyclotron using the ^18^O(p,n)^18^F nuclear reaction, and [^18^F]FLT was synthesized as described elsewhere [27]. Approximately 11–13 MBq [^18^F]FLT was intravenously (*i*.*v*.) injected into the *Ye*-infected and PBS-treated control animals under anesthesia (1.5% isoflurane and 0.8 L/min 100% oxygen), and the mice remained conscious during the 90 min uptake time. The imaging protocol included sequential PET/MR imaging of the PBS-treated control and *Ye-*infected mice on three consecutive days. The animals were examined at 1, 2 and 3 days post infection (*p*.*i*.). Mice were imaged using a small animal PET scanner (Inveon, Siemens Preclinical Solutions, Knoxville, TN, USA) that yielded a spatial resolution of approximately 1.3 mm in the reconstructed images. A 10 min static PET scan was acquired after the uptake time with no attenuation correction. During the PET and MR imaging, the animals were anesthetized with 1.5% isoflurane mixed with 100% oxygen and anesthesia was monitored via measurement of respiratory frequency, and the body temperature was maintained at 37°C using a heating pad placed underneath the mouse. MR imaging was performed on a 7 T small animal MR tomography system (Clinscan, Bruker Biospin MRI, Ettlingen, Germany) to obtain anatomical information directly after the PET scan. The bed with the anesthetized mouse was carefully passed from the PET scanner into the MR without moving the animal. A T2-weighted 3D-space sequence (TE / TR 202 / 2500 ms, image matrix of 137 x 320, slice thickness 0.27 mm) was used for whole-body imaging. The iterative two-dimensional ordered subset expectation maximization algorithm (OSEM2D) reconstructed PET images were normalized to each other according to the injected activity, were manually fused using anatomical landmarks to the respective MR images and analyzed using Inveon Research Workplace software (IRW, Siemens Preclinical Solutions) by drawing regions of interests (ROIs) following anatomical information retrieved from MR images. The results are expressed as the % of the injected dose per cm^3^ (%ID/cc) of tissue. After the final PET scan, all animals were sacrificed by cervical dislocation under deep anesthesia and dissected. The organs were removed, and radioactivity in the tissue samples was quantified with an aliquot of the injected radiotracer in a γ-counter (Wallac 1480 WIZARD 3” Gamma Counter; Perkin Elmer, Waltham, MA, USA) using an energy window between 350 and 650 keV. The results are expressed as % of the injected dose per g (%ID/g) of tissue or as the spleen-to-muscle ratio.

### Histology

The spleens were fixed for 48–72 hours in 4% formalin and embedded in paraffin. For histology, 3–5 μm sections were prepared and stained with hematoxylin and eosin (H&E). The immunohistochemistry was performed on an automated immunostainer (Ventana Medical Systems, Inc.) according to the manufacturer’s protocols for open procedures with slight modifications. All slides were stained with antibodies against Ki67 (catalog number: KI681R06, ready to use, SP6, DCS Innovative Diagnostik-Systeme GmbH u. Co. KG, Hamburg, Germany) and Tk-1 (catalog number: ab57757, dilution 1:500, Abcam plc, 330 Cambridge Science Park, Cambridge, UK). For the retrieval conditions—as part of the automated immunostainer—the program “cc1” for Ki 67 and Tk-1, which used EDTA buffer (ethylenediaminetetraacetic acid buffer) at pH 8 was used. For Ki 67 the retrieval programme “mild” was used with 32 minutes of pretreatment at 90°C to 100°C and “std” for Tk-1 with 64 minutes duration of pretreatment at 90°C to 100°C. Positive and negative controls were used to confirm the adequacy of the staining.

### Statistical Analysis

Statistical significance was determined using one-way analyses of variance (ANOVA) followed by Tukey’s multiple comparison tests. The tests were conducted with Origin 8 software (OriginLab Corporation, Northampton, MA, USA). The data were considered statistically significant at p<0.05. All quantitative data are presented as the mean ± 1 standard deviation (SD).

## Results

### PET/MR imaging of *Ye* with [^18^F]FLT

**Fig 1:**
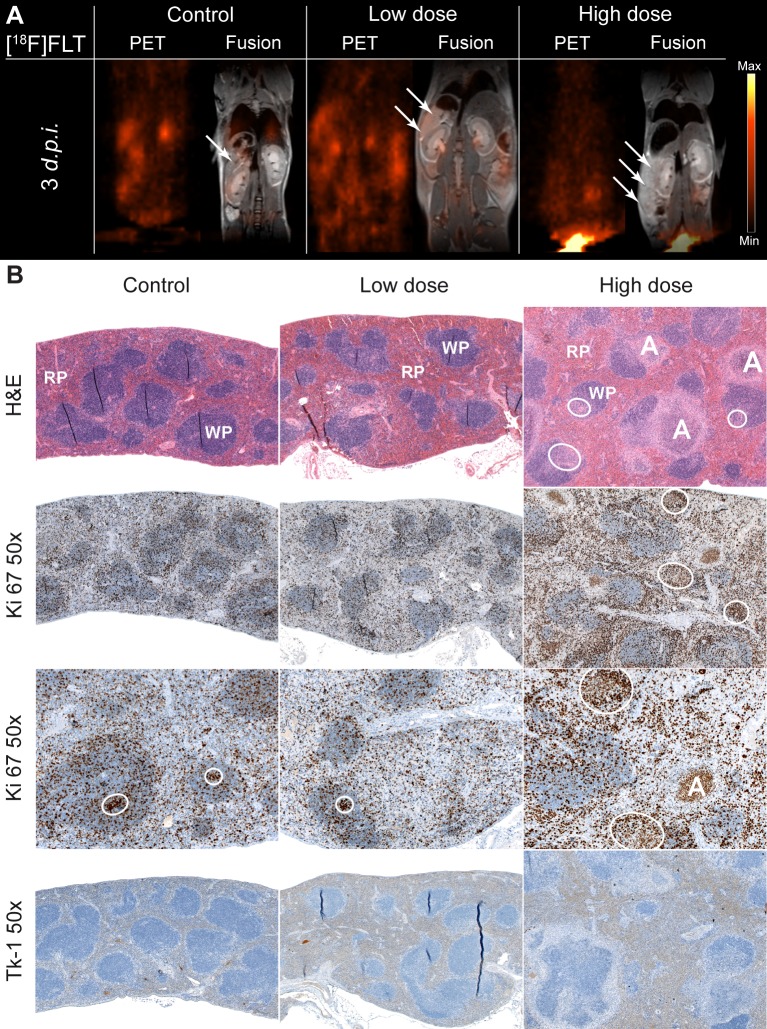
PET imaging of the proliferation in *Ye*-infected mice and the histological findings. (A) Coronal [^18^F]FLT-PET and fused PET and MR images from PBS-treated (n = 3), low- (n = 7) and high dose- (n = 8) infected mice 3 days *p*.*i*. The arrows indicate the positions of the spleens, which showed no increased uptake of [^18^F]FLT in *Ye* infected animals compared to PBS-treated controls. (B) Immunohistochemical stainings of spleen tissue sections from the PBS-treated, low dose-, and high dose-infected mice 3 days *p*.*i*. Sections were stained for H&E, Ki 67 and Tk-1.The encircled areas indicate germinal centers. A: abscess, RP: red pulp, WP: white pulp. Data are representative of 6 analyzed mice.

The clinical PET tracer of cell proliferation [^18^F]FLT was used to image systemic *Ye* infections in mice. As we demonstrated in a previous study, the bacteria mainly localize to the spleen, liver, lung and bone marrow upon systemic infection in mice, [28]. Here, the mice were infected with either 5 x 10^4^ (high-dose) or 1 x 10^3^ (low-dose) *Ye* and examined in PET and MRI one to three days *p*.*i*. *In vivo* evaluations of the tracer revealed similar uptake patterns of [^18^F]FLT in the spleens in the low- and high-dose groups throughout the course of infection, which demonstrated that tracer uptake was independent of the infection level. More precisely, the [^18^F]FLT uptake in the spleens was comparable to that of the background signal in the muscle tissue (Fig 1A, Table 1). Quantification of the [^18^F]FLT uptake of various organs revealed results similar to those obtained in the *ex vivo* biodistribution study and confirmed the PET results (Table 2).

**Table 1:** *In vivo* biodistributions of [^18^F]FLT in the PBS-treated control, low-dose and high-dose Ye-infected mice. The averages and standard deviations are given for indicated organs based on the ROIs of the PET images. Static 10 min PET scans of PBS-treated, low-dose- and high-dose infected mice were acquired 1, 2 and 3 days *p*.*i*. The errors indicate one standard deviation. 11 to 13 MBq of [^18^F]FLT were administered to each mouse.

[^18^F]FLT, %ID/cc (± 1 SD)						
Group	Acquisition	Spleen	Liver	Spine	Brain	Muscle
PBS	1	0.18 ± 0.01	0.17 ± 0.01	0.27 ± 0.02	0.06 ± 0	0.13 ± 0.02
	2	0.43 ± 0.2	0.21 ± 0.02	0.4 ± 0.2	0.08 ± 0.02	0.17 ± 0.02
(n = 3)						
	3	0.55 ± 0.29	0.2 ± 0.07	0.36 ± 0.13	0.06 ± 0.01	0.19 ± 0.05
Low dose	1	0.26 ± 0.09	0.2 ± 0.07	0.25 ± 0.06	0.09 ± 0.03	0.14 ± 0.05
	2	0.33 ± 0.08	0.24 ± 0.05	0.29 ± 0.06	0.08 ± 0.02	0.19 ± 0.04
(n = 7)						
	3	0.64 ± 0.23	0.34 ± 0.24	0.38 ± 0.11	0.09 ± 0.06	0.24 ± 0.16
High dose	1	0.17 ± 0.07	0.17 ± 0.07	0.15 ± 0.04	0.09 ± 0.02	0.11 ± 0.04
(n = 8)	2	0.22 ± 0.04	0.21 ± 0.05	0.19 ± 0.05	0.14 ± 0.03	0.17 ± 0.04
	3	0.37 ± 0.18	0.36 ± 0.14	0.42 ± 0.16	0.21 ± 0.06	0.29 ± 0.09

**Table 2:** *Ex vivo* biodistribution of selected organs of PBS-treated control, low-dose and high-dose-infected animals at day 3 *p*.*i*. No increased [^18^F]FLT uptake was observed in any of the tested organs. The errors indicate one standard deviation or the spleen-to-muscle ratios. 11 to 13 MBq of [^18^F]FLT were administered to each mouse.

[^18^F]FLT	PBS-treated mice	low-dose infected mice	high-dose infected mice
%ID/g (± 1 SD)			
Organ	(n = 3)	(n = 7)	(n = 8)
Blood	0,19 ± 0,04	0,29 ± 0,20	0,38 ± 0,17
Spleen	0,80 ± 0,36	1,10 ± 0,59	0,76 ± 0,56
Liver	0,34 ± 0,14	0,40 ± 0,22	0,58 ± 0,35
Bone marrow	0,68 ± 0,24	0,53 ± 0,18	0,80 ± 0,23
Brain	0,08 ± 0,04	0,10 ± 0,05	0,29 ± 0,24
Muscle	0,24 ± 0,03	0,42 ± 0,22	0,56 ± 0,19
Spleen/muscle	3,28 ± 1,03	4,10 ± 3,89	1,40 ± 0,90

### Immunohistochemistry

To confirm the PET results, sections of the spleens from all groups of mice were analyzed via immunohistochemistry. Macroscopically, the PBS-treated and low-dose *Ye*-infected mice had normal-sized spleens, whereas the spleens of the high-dose infected animals were enlarged. Additionally, the spleens of the high-dose infected mice exhibited lymphoid hyperplasia with germinal centers, multiple abscesses and necrosis as detected by H&E staining (Fig 1B). The proliferation rate was analyzed with Ki 67 in the spleens. Normal distribution of the proliferation (white pulp vs red pulp) was detected in the spleens of the PBS-treated and low-dose infected mice, and no difference between these two groups was observed. In contrast, the spleens of the high-dose infected mice revealed increased Ki 67 expression in the hyperplastic germinal center and in the neutrophils (based on nuclear morphology) located in the abscesses. Moreover, the low [^18^F]FLT uptake presented in PET imaging corresponded with thymidine kinase 1 (Tk-1) staining, which revealed no Tk-1 staining in the spleens of infected or PBS-treated mice (Fig 1B).

## Discussion

The PET tracer [^18^F]FLT acts as a surrogate marker for cell proliferation and is primarily used for the detection of cancer cells [29]. [^18^F]FLT has received little attention as a possible tracer for inflammation and the diagnosis of infectious diseases [17–18]. In the salvage pathway, [^18^F]FLT is transported across the cell membrane by nucleoside carrier proteins, and is phosphorylated by S-phase-specific thymidine kinase 1 (Tk-1) up-regulated in the late G1-S phase of the cell cycle of proliferating cells. This leads to trapping of the tracer in the cytosol as monophosphate without DNA incorporation and its subsequent availability as a substrate for cytoplasmic Tk-1 [29–30]. A second mechanism for DNA synthesis in proliferating cells has been identified as the *de novo* pathway. In this pathway deoxyuridine monophosphate is converted into thymidine monophosphate by the enzyme thymidylate synthase. Thymidine monophosphate is subsequently phosphorylated and incorporated into the DNA. Both pathways are complementary to each other and are able to provide sufficient thymidine for DNA synthesis during proliferation.

In nuclear medicine and molecular imaging, considerable efforts have been made to detect pathogens by using new radiopharmaceuticals based on small molecules, peptides, intact antibodies or their fragments [4, 31–35]. Antibody-guided immunoPET has been reported to specifically visualize SIV *in vivo* in an animal model [35]. We recently applied this powerful tool for the specific detection of the fungal pathogen *Aspergillus fumigatus* and bacterial pathogen *Ye* [15, 36]. The advantage of immunoPET over conventional small molecule PET tracers, such as [^18^F]FLT, is that immunoPET enables the pathogen-specific detection of infections and the discrimination of infections from general inflammatory responses.

Only a very limited number of studies have investigated the proliferation marker [^18^F]FLT as a means of imaging infectious diseases in animal models. However, [^18^F]FLT-PET was used in an animal model of the parasitic disease alveolar echinococcosis by Porot et al. [37] and by Rolle et al. [38]. Although both groups showed elevated uptake of [^18^F]FLT in parasitized tissue *in vitro*, contradictory findings were presented for *in vivo* imaging studies. Porot et al. showed no uptake of [^18^F]FLT in their animal model of the disease, whereas Rolle et al. showed elevated uptake of [^18^F]FLT in parasitized tissues, which was likely caused by inflammation at the site of infection [37–38]. Successful detection with [^18^F]FLT of localized bacterial infections has also been reported in an animal model, where *Staphylococcus aureus* was implanted into the tibia of rabbits [22], and in a mouse model of *Salmonella typhimurium* infection in the thigh muscle [23].

In our imaging studies, the high-dose *Ye-*infected mice exhibited no uptake of [^18^F]FLT, whereas the immunohistochemical staining with Ki 67 revealed proliferation in the hyperplastic germinal centers. Our results are consistent with tumor imaging studies performed with [^18^F]FLT that have demonstrated varying degrees of correlation between the uptake of the proliferation tracer and the immunohistochemical marker Ki 67 [8, 39]. Our PET findings were confirmed with additional Tk-1 immunohistochemical staining that revealed no Tk-1 staining in the spleens of the infected or PBS-treated mice. The intensity of Tk-1 in *ex vivo* immunohistochemistry staining is dependent on the DNA synthesis pathway utilised in the different tissues investigated. As Tk-1 expression is correlated with the salvage pathway, no Tk-1 staining can be observed in proliferating tissues that use the *de novo* pathway. However, Ki 67 is an antigen, which is expressed during all active phases of the cell cycle (G1, S, G2, and M) of proliferating cells and is independent of the pathway used for DNA synthesis. The antigen is not expressed during the G0 phase of resting or quiescent cells [40]. It might be considered unfortunate that Ki 67 has historically been used as the gold standard for validation of [^18^F]FLT-PET imaging, as evidenced by the conflicting correlations between Ki 67 and the PET marker [^18^F]FLT as stated by McKinley et al. [8]. Based on our findings, we conclude that the proliferation marker [^18^F]FLT is not suitable for the *in vivo* imaging of *Ye* infections.
